# An Increased Abundance of Tumor-Infiltrating Regulatory T Cells Is Correlated with the Progression and Prognosis of Pancreatic Ductal Adenocarcinoma

**DOI:** 10.1371/journal.pone.0091551

**Published:** 2014-03-17

**Authors:** Yichen Tang, Xuejun Xu, Shixiang Guo, Chaobin Zhang, Yan Tang, Yi Tian, Bing Ni, Binfeng Lu, Huaizhi Wang

**Affiliations:** 1 Institute of Hepatopancreatobiliary Surgery, Southwest Hospital, Third Military Medical University, Chongqing, China; 2 Institute of Immunology PLA, Third Military Medical University, Chongqing, China; 3 Department of Immunology, University of Pittsburgh School of Medicine, Pittsburgh, United States of America; Mie University Graduate School of Medicine, Japan

## Abstract

CD4^+^CD25^+^Foxp3^+^ regulatory T cells (Tregs) can inhibit cytotoxic responses. Though several studies have analyzed Treg frequency in the peripheral blood mononuclear cells (PBMCs) of pancreatic ductal adenocarcinoma (PDA) patients using flow cytometry (FCM), few studies have examined how intratumoral Tregs might contribute to immunosuppression in the tumor microenvironment. Thus, the potential role of intratumoral Tregs in PDA patients remains to be elucidated. In this study, we found that the percentages of Tregs, CD4^+^ T cells and CD8^+^ T cells were all increased significantly in tumor tissue compared to control pancreatic tissue, as assessed via FCM, whereas the percentages of these cell types in PBMCs did not differ between PDA patients and healthy volunteers. The percentages of CD8^+^ T cells in tumors were significantly lower than in PDA patient PBMCs. In addition, the relative numbers of CD4^+^CD25^+^Foxp3^+^ Tregs and CD8^+^ T cells were negatively correlated in the tissue of PDA patients, and the abundance of Tregs was significantly correlated with tumor differentiation. Additionally, Foxp3^+^ T cells were observed more frequently in juxtatumoral stroma (immediately adjacent to the tumor epithelial cells). Patients showing an increased prevalence of Foxp3^+^ T cells had a poorer prognosis, which was an independent factor for patient survival. These results suggest that Tregs may promote PDA progression by inhibiting the antitumor immunity of CD8^+^ T cells at local intratumoral sites. Moreover, a high proportion of Tregs in tumor tissues may reflect suppressed antitumor immunity.

## Introduction

Pancreatic cancer is the fourth leading cause of cancer deaths in the USA and leads to an estimated 227,000 annual deaths worldwide [1]. Pancreatic ductal adenocarcinomas (PDAs) evolve through non-invasive precursor lesions, typically through pancreatic intraepithelial neoplasias. Early-stage pancreatic cancer is usually clinically silent, and the disease only becomes apparent after the tumor invades the surrounding tissues or metastasizes to distant organs. Most people who present with symptoms attributable to pancreatic cancer exhibit advanced disease [2]. As early PDA detection is difficult and there are few therapeutic strategies available to treat advanced tumors, there is a pressing need to develop novel therapies for advanced PDA.

Immunotherapy is an attractive strategy for cancer treatment because the immune response specificity may circumvent many side effects associated with the currently available clinical options [3]. Effective CD8^+^ T cells that mediate cytotoxic killing may play a crucial role in the antitumor immune reaction by releasing granules such as perforin and granzymes [4]. Recently, several studies have shown that tumor-infiltrating CD8^+^ T cells prolong survival in patients with cervical carcinoma and ovarian Cancer [5], [6]. Moreover, intratumoral CD8^+^ T cells abundance was positively correlated with a good survival in PDA patients [7]. Therefore, tumor-infiltrating CD8^+^ T cells are believed to be a favorable prognostic indicator in a variety of tumors. However, cancer cells protect themselves from co-stimulatory molecules on the cell surface and via the secretion of cytokines, such as IL-10 and TGF-β, to alter the tumor microenvironment and diminish the antitumor response efficacy [8]. IL-10 and TGF-β are the most important cytokines for the differentiation of naive T cells into Tregs [9]. In contrast, Tregs have previously been reported to reduce the effects of immune T cells, such as CD8^+^ T cells, or to suppress T cell functions, leading tumor cells to escape immune surveillance [10], [11].

The immune system constitutes an important part of the tumor microenvironment, and it is thought to be critical for cancer development and progression. Many studies have suggested that Tregs are major players in tumor immune suppression [12] and that they represent the main obstacle to successful tumor immunotherapy [13], [14]. Tregs accumulate in tumors and in the peripheral blood of patients with cancer [15]–[17]. Increasingly, studies have confirmed that Tregs are recruited to tumor sites, where they suppress antitumor cytotoxic responses [18]–[20]. It has been shown that the numbers of CD4^+^CD25^+^Foxp3^+^ Tregs are increased in the peripheral blood mononuclear cells (PBMCs) and draining lymph nodes of human colon cancer patients, and these Tregs are capable of suppressing antigen-specific CD4^+^ T cells [21]. The surgical removal of colon cancer reduces the Treg population and restores the antigen-specific T cell activity of CD4^+^ T cells [22]. Immunoregulatory mechanisms present in the tumor microenvironment, including in the liver [23], breasts [24], [25] and ovaries [26], may contribute to tumor outgrowth. Immunohistochemical (IHC) studies have revealed the presence of Foxp3^+^ T cells in PDA tissue and shown their correlation with a poor clinical prognosis [27], [28]. Several studies have analyzed the frequency of Tregs using FCM in the PBMCs of PDA patients [29], [30]. However, the proportion of T cell subtypes in PDA tissue where the T cells would function on tumor cells, have not been elucidated by FCM. In addition, animal models show that Tregs actively infiltrate the stromal compartment of pancreatic intraepithelial neoplasias and PDAs, even in the earliest tumor development stages, and display local immunosuppression [31]. Therefore, it is necessary to assess the Treg distribution details in PDA tissue to investigate the mechanisms underlying the potential Treg effects in PDA.

This study was therefore designed to examine the role of tumor-infiltrating Tregs that can potentially affect the tumor-specific T-cell response in patients with PDA. The proportions of CD4^+^/CD8^+^ T cells and Tregs in tumor-infiltrating lymphocytes (TILs) and in the PBMCs of PDA patients were analyzed using FCM. To analyze the correlation between Treg abundance and the abundance of CD8^+^ T cells or CD4^+^ T cells in the PDA microenvironment, we also investigated the relationship between the proportion of tumor-infiltrating CD8^+^ T cells or Tregs and clinicopathologic characteristics. After verifying the expression of CD4^+^ and Foxp3^+^ T cells in PDA tissue via IHC, the relationship of CD4 and Foxp3 expression with PDA patient survival was analyzed. These results suggest that Tregs may inhibit the antitumor immunity of CD8^+^ T cells in PDA and be correlated with poor PDA differentiation.

## Materials and Methods

### Ethics Statement

This study was approved by the ethical committee of Southwest Hospital. All patients provided written informed consent.

### General information

In total, 228 patients with PDA and 15 healthy controls treated at Southwest Hospital between January 2007 and January 2012 were enrolled in this study. Of these patients, 143 were male, and 85 were female. Their average age was 57.13±12.16 years. Forty-five patients were analyzed using FCM, and 183 patients were analyzed using only IHC. The patients' demographics and tumor characteristics, including the tumor stage, lymph node metastasis, tumor size and degree of differentiation, are shown in **Table 1**. None of the patients received radiotherapy, chemotherapy or other medical interventions. The 15 controls included 10 male and 5 female individuals with an average age of 55.86±9.10 years. The patients and controls were matched for both sex and age.

**Table:** **Table 1.** Patient demographic and tumor characteristics.

Clinicopathologic characteristics	Results
Total cases	228
Age(years)	
Mean (±SD)	57.13±12.16
Median (range)	66 (33–78)
Gender	
Male	147
Female	81
Tumor size (mm)	
Mean (±SD)	42.7±15.3
Median (range)	37 (18–109)
Pathologic tumor status	
pT1	41
pT2	80
pT3	92
pT4	15
Regional lymph node status	
N0	178
N1	50
Distant metastasis status	
M0	213
M1	15
Stage	
I+II	187
III+IV	41
Tumor differentiation	
Well	48
Moderate	106
Poor	51
Other histologic type	23
Perineural invasion(absent/present)	171/57
Vascular invasion(absent/present)	195/33

Note: Classified according to International Union Against Cancer tumor-node-metastasis classification.

### Blood and tissue samples

During the study, 45 additional patients and 15 healthy volunteers were enrolled. PBMCs were isolated via Lymphoprep (Roche, Basel, Switzerland) density gradient centrifugation. Tumor specimens and normal pancreatic tissue specimens were obtained from the PDAs of patients or at the distal ends of the surgical margin; the non-tumor portions were verified to be free of cancer involvement through microscopic examination. Fresh tumor samples were collected during surgery and chopped into small pieces using a razor blade in RPMI 1640 medium. These tissues were mixed with 1 mg/ml collagenase-IV (Sigma-Aldrich, St. Louis, MO) and 10 mg/ml DNase I (Roche, Basel, Switzerland) in Hank's buffered salt solution for 30 minutes at 37°C and then mechanically dissociated with a MACS Dissociator (Miltenyi Biotec, Bergisch Gladbach, Germany), followed by filtration through a 70-µm nylon mesh. Single-cell suspensions were separated with Lymphoprep (Roche, Basel, Switzerland). The tumor tissue samples from 183 patients were fixed in 4% paraformaldehyde and embedded in paraffin for histopathological analysis.

### FCM

Cells from tissue samples and PBMC suspensions were incubated in 1 ml of PBS with 1 µl per sample of LIVE/DEAD Fixable Red Dead Stain (Invitrogen) for 30 minutes on ice in the dark. The cells were washed, re-suspended and stained with anti-human CD3 APC-eFluor780 (47-0036, eBioscience), CD4-FITC (11-0048-41, eBioscience), CD8 PerCP-Cy5.5 (45-0088, eBioscience) and CD25-PE antibodies (12-0259-41, eBioscience). After 30 minutes on ice, the samples were washed and fixed in PBS containing 1% paraformaldehyde (PFA; Sigma-Aldrich). The cells were washed and resuspended with 1 ml of permeabilization buffer (00-5523-00 Foxp3 Staining Buffer Set eBioscience) for one hour and then washed and incubated for 30 minutes in permeabilization buffer containing APC-conjugated antibodies against Foxp3 (12-4776-41, eBioscience). Isotype controls were used to enable accurate compensation and to confirm antibody specificity. The stained cells were analyzed via FCM using a FACS Calibur flow cytometer (BD Bioscience PharMingen, San Jose, CA, USA) equipped with CellQuest software (BD Bioscience PharMingen, San Jose, CA, USA).

### Immunohistochemistry

Consecutive 5-µm sections were cut from the paraffin-embedded samples. For antigen retrieval, the de-paraffinized sections were boiled for 2.5 minutes in citrate buffer, pH 6.0. Endogenous peroxidase activity was blocked through incubation with a 3% hydrogen peroxide solution for 20 minutes at room temperature. Primary antibody dilutions were prepared as follows: mouse anti-human-Foxp3 (monoclonal, 1∶100 dilution, Biosynthesis Biotech, Abcam), rabbit anti-human-CD8 (monoclonal, 1∶100 dilution, RMA-0514, Maixin, China) and rabbit anti-human-CD4 (monoclonal, 1∶100 dilution, Biosynthesis Biotech, Maixin, China) at 4°C overnight, followed by incubation with a horseradish peroxidase-labeled polymer conjugated to the secondary goat anti-mouse/rabbit antibody (KIT-9710; Maixin, China) at 37°C for 30 minutes. Finally, the signal was developed with 3,3′-diaminobenzidine (DAB-2031; Maixin, China), and all slides were counterstained with hematoxylin. Negative controls were treated with PBS instead of the primary antibodies.

### Scoring system for immunohistochemistry analysis

The FoxP3 and CD4 distributions were observed under an optical microscope (BX51; Olympus, Tokyo, Japan) by independent pathologists who were blinded to the clinical outcome, and positive staining was quantitatively evaluated using Image-Pro Plus 5.1 software (Media Cybernetics, Inc., Silver Spring, Md., USA) in at least five different high-power fields (20× objective and 10× eyepiece). The number of Foxp3^+^, CD4^+^ lymphocytes was calculated for each field, and the averages were compared.

### Data processing

All data are expressed as the median values and ranges. Comparisons between the two groups were assessed using Student's t test. Correlations between the parameters were assessed through Pearson correlation analysis and linear regression analysis. The X^2^ test or Fisher's exact test was used to compare categorical variables when associating the Treg and CD8^+^ T cell prevalences with various clinicopathologic variables. Survival rates were calculated using the Kaplan-Meier method. Survival analysis began on the day of surgical resection and ended on the day of death or at the end of observations. Differences between survival curves were analyzed using log-rank tests. Multivariate analyses were performed using the Cox proportional hazards regression model. Statistical analyses were conducted using Graph Pad Prism 5.0 software. *P* values less than 0.05 were considered statistically significant (* *P*<0.05; ** *P*<0.01; *** *P*<0.001).

## Results

### Up-regulation of CD4^+^T, CD8^+^T and Treg cells in PDA tissue

**Figure 1:**
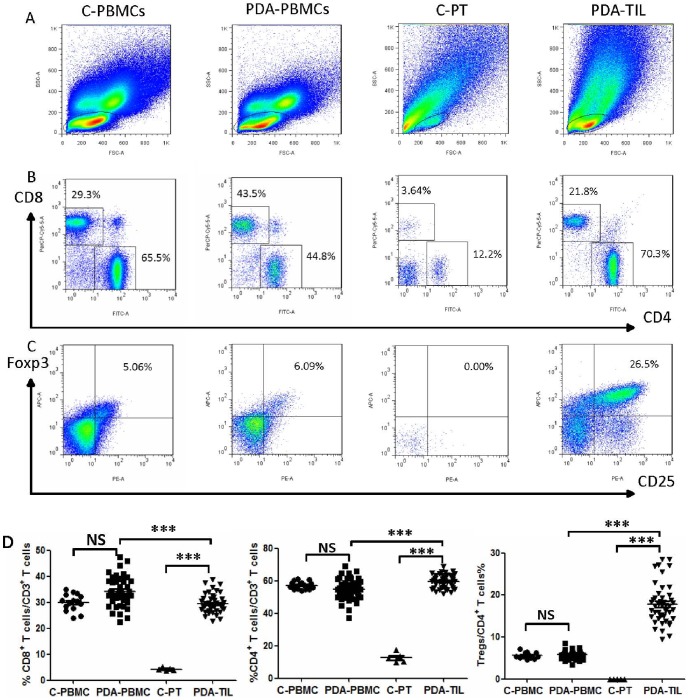
Flow cytometric analysis of the T cell subtype proportions. The proportions of CD4^+^ T cells, CD8^+^ T cells and Tregs in PBMCs and in pancreatic tissue lymphocytes or tumor-infiltrating lymphocytes from healthy control and PDA patients were analyzed via FCM. (A) Lymphocyte dot plots. The gate for lymphocytes is indicated. (B) CD4^+^ and CD8^+^ T cells were defined based on CD4^+^CD8^−^ and CD8^+^CD4^−^ gating of CD3^+^ T cells. (C) Dot plots of Foxp3^+^CD25^+^ (Treg) cells based on the gating of CD4^+^ T cells. (D) Statistical analyses of the CD8^+^ T cell, CD4+ T cell and Treg percentages in the indicated groups. C-PBMCs, control peripheral blood mononuclear cells; PDA-PBMCs, PBMCs of PDA; C-PTL, control pancreatic tissue lymphocytes; PDA-TIL, tumor-infiltrating lymphocytes of PDA. Comparisons between the two groups were assessed using Student's t test. NS, not significant; *** *P*<0.001.

The proportions of T cells in PBMCs and in pancreatic tissue or PDA tissue collected from 15 healthy controls and 45 PDA patients (28 males and 17 females with a mean age of 57 years) were analyzed via FCM. The results showed that the percentages of CD8^+^ T cells (PDA-PBMC *vs.* C-PBMC: 34.28±0.8431% *vs.* 30.94±0.8405%), CD4^+^ T cells (PDA-PBMC *vs.* C-PBMC: 54.51±0.9725% *vs.* 57.17±0.6233%) and Tregs (PDA-PBMC *vs.* C-PBMC: 5.755±0.145% *vs.* 5.772±0.2105%) in PBMCs were not markedly different between PDA patients and healthy volunteers (**Figure 1D**). We also observed that the proportions of CD4^+^ T cells (PDA-TIL *vs.* C-PT: 59.47±0.5974% *vs.* 12.6±1.343%) and Tregs (PDA-TIL *vs.* C-PT: 17.8±0.7075% *vs.* 0.01±0.005%) in TILs were markedly increased compared to C-PTLs (both *P*<0.001) (**Figure 1B, 1C and 1D**). However, the proportion of CD8^+^ T cells in the TILs of PDAs was significantly higher than that in control pancreatic tissue lymphocytes (C-PTLs) (29.48±0.5353% *vs.* 4.282±0.2738%, *P*<0.001) (**Figure 1B and 1D**). Taken together, these data demonstrate an enrichment of CD8^+^ T cells, CD4^+^ T cells and Tregs in PDA tissue relative to healthy pancreatic tissue, suggesting that T cell immunity and Tregs may be playing an important role in the development and progression of PDA.

When the relative frequency of T subtypes was compared between the PBMCs and TILs of PDA patients, we found that the percentages of CD4^+^ T cells and Tregs in TILs were increased compared to the percentages in PDA PBMCs (both *P*<0.001) (**Figure. 1B, 1C and 1D**). However, the percentage of CD8^+^ T cells was down-regulated in PDA TILs (PDA TILs vs. PDA PBMCs: 29.48±0.5353% vs. 34.28±0.8431%, *P*<0.001) (**Figure 1B and 1D**). In contrast, the proportions of CD8^+^T cells, CD4^+^T cells and Tregs in the pancreatic tissues of healthy controls were much lower than in PBMCs (**Figure 1B, 1C and 1D**).

### Association of tumor-infiltrating Tregs with clinicopathologic characteristics

**Figure 2:**
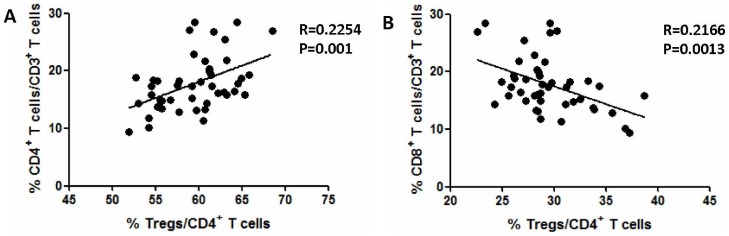
Correlation between tumor-infiltrating Tregs and CD4+ T cells or CD8+ T cells in PDA tissue. The correlation of the tumor-infiltrating Treg frequency with that of tumor-infiltrating CD4^+^ T cells or tumor-infiltrating CD8^+^ T cells was analyzed. Correlations between the parameters were assessed through Pearson correlation analysis.

We investigated the Treg role in the PDA microenvironment by correlating the proportions of Tregs and CD4^+^ T cells or CD8^+^ T cells in the tumor tissues of PDA patients. We found that the relative intratumoral Treg abundance was positively correlated with the relative intratumoral CD4^+^ T cell abundance (R = 0.2254, *P* = 0.001) (**Figure 2A**) but negatively correlated with the relative intratumoral CD8^+^ T cell abundance (R = 0.2166, *P* = 0.0013) (**Figure 2B**). These data suggest that Tregs may promote PDA progression by inhibiting the antitumor immunity of CD8^+^ T cells at local intratumoral sites.

To explore the possible roles of tumor-infiltrating Tregs and CD8^+^ T cells in the development of PDA, we analyzed the correlation between the proportion of tumor-infiltrating Tregs or CD8^+^ T cells and the clinicopathologic PDA characteristics. PDA patients were divided into two groups based on the median value obtained for either tumor-infiltrating Tregs (17.37% in CD4^+^ T cells) or tumor-infiltrating CD8^+^ T cells (28.69% in CD3^+^ T cells) (**Table 2**). The results indicated that the proportion of intratumoral CD8^+^ T cells was not correlated with any clinicopathologic characteristics. The number of tumor-infiltrating Tregs showed a significant positive correlation with tumor differentiation (*P*<0.001) but was not correlated with tumor pathologic metastasis or tumor micro-vascular invasion. Therefore, increased numbers of tumor-infiltrating Tregs may be correlated with the microenvironment and PDA differentiation.

**Table 2:** Correlations between tumor-infiltrating Tregs or CD8^+^ T cells and the clinicopathologic characteristics of 45 patients with PDA.

Characteristics	Intratumor CD8^+^ T cells MV = 28.69%			Intratumor Tregs MV = 17.37%		
	Low(n = 23)	High(n = 22)	*P*	Low(n = 23)	High(n = 22)	*P*
Sex						
Male	15(65.2)	13(59.1)		16(69.6)	12(54.5)	
Female	8(34.8)	9(40.9)	1.00^1^	7(30.4)	10(45.4)	0.365^1^
Mean age ± SD, years	54.67±10.42	57.17±10.54	0.429^2^	56.04±10.91	55.95±10.18	0.978^2^
Pathologic tumor status						
pT1	5(21.7)	3(13.6)		6(26.1)	2(9.1)	
pT2	7(30.4)	5(22.7)		7(30.4)	5(22.7)	
pT3	9(39.1)	12(54.5)		8(34.8)	13(59.1)	
pT4	2(8.7)	2(9.1)	0.558^1^	2(8.7)	2(9.1)	0.320^1^
Pathologic metastasis status						
M0	20(87.0)	20(90.9)		21(91.3)	19(86.4)	
M1	3(13.0)	2(9.1)	0.872^1^	2(8.7)	3(13.6)	0.598^1^
Pathologic node status						
N0	19(82.6)	18(81.8)		20(87.0)	17(77.3)	
N1	4(17.3)	4(18.1)	1.00^1^	3(13.0)	5(22.7)	0.243^1^
Stage						
I+II	17(73.9)	17(77.3)		17(73.9)	17(77.3)	
III+IV	6(26.1)	5(22.7)	0.793^1^	6(26.1)	5(22.7)	0.793^1^
Tumor Grade						
Well	1(4.3)	8(36.4)		9(39.1)	0(0)	
Moderate	13(56.5)	10(45.5)		13(56.5)	10(45.5)	
Poor	9(39.1)	4(18.2)	0.061^1^	1(4.3)	12(54.5)	<0.001^1^
Vascular invasion						
Present	6(26.1)	3(13.6)		4(17.4)	5(22.7)	
Absent	17(73.9)	19(86.4)	0.459^1^	19(82.6)	17(77.3)	0.722^1^
Perineural invasion						
Present	8(34.8)	6(27.2)		7(30.4)	7(31.8)	
Absent	15(65.2)	16(72.7)	0.749^1^	16(69.6)	15(68.2)	1.00^1^

1 χ^2^ test or Fisher's exact test;

2 Student's t test. MV = median value.

Classified according to International Union Against Cancer tumor-node-metastasis classification.

### Foxp3^+^ cells were observed more frequently in tumor tissue

**Figure 3:**
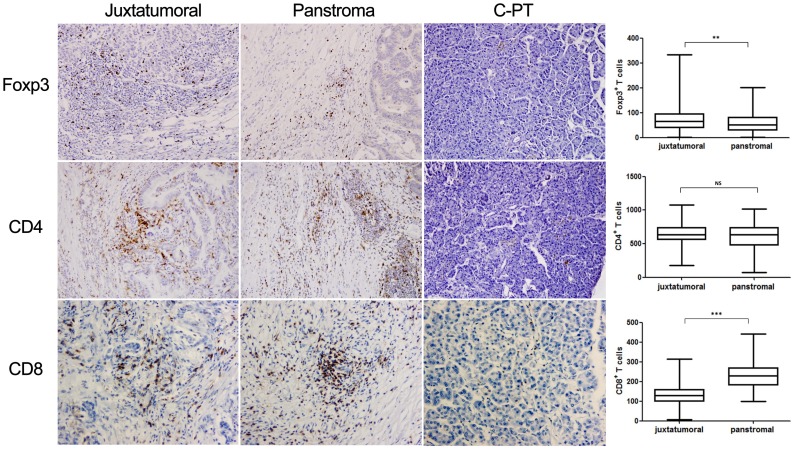
IHC analysis of paraffin-embedded tumor sections from patients with PDA or control pancreatic tissue. Magnification: 200×. Comparisons of FoxP3^+^ cell, CD8^+^ cell and CD4^+^ cell infiltrates between the juxtatumoral stroma and the panstroma. IHC analyzed the expression of Foxp3, CD4 and CD8 and statistical analysis the frequencies in juxtatumoral stroma and in panstroma. Comparisons between the two groups were assessed using Student's t test. NS, not significant; ** *P*<0.01, *** *P*<0.001.

The above results indicated that the percentages of CD4^+^CD25^+^FoxP3^+^ Tregs were increased, as assessed via FCM. To confirm these findings and examine the correlation between tumor-infiltrating Foxp3^+^ T cells and the clinicopathologic PDA characteristics, we examined the Foxp3+ T cell abundance in PDA tissues from 160 patients and five control specimens (Figure 3). Though obvious CD4, CD8 and Foxp3 expression were not observed in normal pancreatic tissue, Foxp3 was strongly expressed in the tumor stroma, with more Foxp3^+^ cells have been observed in the PDA juxtatumoral stroma than in the PDA panstroma (*P* = 0.0043, R = 0.02217) (**Figure 3**). We next analyzed the distribution of CD8^+^ cells within the stromal of PDA and found that the CD8^+^ cells were significantly reduced in the juxtatumoral stroma compared with the panstromal areas (*P*<0.0001, R = 0.4802) (**Figure 3**). Interestingly, although many CD4^+^ cells had infiltrated the PDA juxtatumoral stroma and the PDA panstroma, the frequency of CD4^+^ cells did not significantly differ between the local sites (*P* = 0.1264, R = 0.0064) (**Figure 3**). To determine if the proportion of intratumoral Foxp3+ cells correlated with any clinicopathological characteristic, the 160 patients were split into two groups based on the median percentage of intratumoral Foxp3+ cells. Of the examined characteristics, only the intratumoral abundance of Foxp3^+^ T cells was correlated with tumor differentiation, the pathologic tumor status, tumor stage and lymphatic invasion (**Table S1**).

### Prognostic significance of Foxp3^+^, CD8^+^ and CD4^+^ cells in the PDA microenvironment

**Figure 4:**
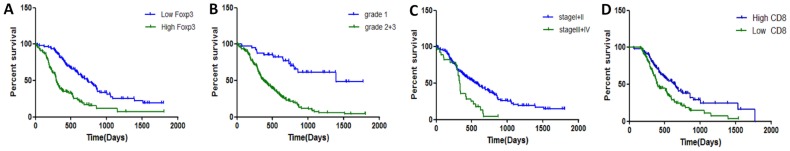
Survival curves for univariate analyses. (A) The survival rates of PDA patients with a high or low proportion of intratumoral Foxp3^+^ cells were estimated by the Kaplan-Meier method. The log-rank test was applied to compare the two groups. (B) Survival rates for patients with different tumor histological grades were estimated by the Kaplan-Meier method. (C) Survival rates for patients with the indicated TNM stages were estimated by the Kaplan-Meier method. (D) Survival rates for patients with a high or low proportion of intratumoral CD8^+^ cells were estimated by the Kaplan-Meier method.

Kaplan-Meier univariate survival analysis showed that the overall survival rate was significantly reduced in patients showing an increased intratumoral Foxp3^+^ cell density compared to those with a lower Foxp3^+^ cell density, as determined through IHC assays (*P*<0.0001, **Figure 4A** and **Table 3**). Thus, patients with a high number of tumor-infiltrating Foxp3^+^ cells display an unfavorable prognosis. We also found a higher number of tumor-infiltrating CD8^+^ cells were associated with an improved survival (*P* = 0.0016, **Figure 4D** and **Table 3**). In contrast, the overall survival rates of patients with low versus high numbers of CD4^+^ cells did not significantly differ (*P* = 0.8082, **Table 3**). A multivariate Cox proportional hazards model analysis was performed, and variables that were associated with survival based on univariate analysis were adopted as covariates (**Table 3**). The multivariate analysis indicated that the intratumoral density of Foxp3^+^ cells (hazard ratio (HR) = 0.3162, *P*<0.0001), histologic grade (HR = 0.3189, *P* = 0.0018, **Figure 4B**) and TNM stage (HR = 0.2185, P = 0.0162, **Figure 4C**) were independent prognostic factors for the PDA patient overall survival. These results suggest that increased intratumoral Foxp3^+^ cell numbers are associated with PDA progression and may serve as an independent predictor of poor survival in PDA patients.

**Table 3:** Univariate and multivariate analyses of the association of clinicopathologic characteristics with overall survival in 160 patients with PDA.

			Univariate analysis			Multivariate analysis		
Variables	Categories	MST (95%CI)	Overall survival			Overall survival		
			HR	95% CI	*P*	HR	95% CI	*P*
Age	≤58	530(374.4–685.6)	0.8218	0.5765–1.2653	0.2152	1.327	0.7576–1.9519	0.3574
	>58 years	428(293.3–562.7)						
Sex	Male	525(388.2–611.8)	0.8378	0.5854–1.2416	0.3316	0.8546	0.5916–1.2753	0.4361
	Female	400(268.2–531.8)						
Histologic grade	Well	587(475.0–699.0)	0.3427	0.2416–0.4741	<0.0001	0.3453	0.2636–1.5325	0.0025*
	Moderate+Poor	280(204.7–355.3)						
Tumor status	T1+pT2+pT3	472(459.9–635.7)	0.3931	0.1831–1.0359	0.0413	0.3135	0.1364–0.5935	0.0743
	pT4	305(200.9–443.1)						
Node status	pN0	471(458.0–628.0)	0.6925	0.5855–1.035	0.0910	0.7493	0.5143–0.9463	0.3637
	pN1	374(327.0–518.7)						
Metastasis	pM0	448(485.3–600.6)	0.3642	0.1546–0.8613	0.0215	0.3169	0.1424–0.7328	0.2426
status	pM1	334(186.5–451.9)						
TNM stage	I–II	472(374.4–486.8)	0.4143	0.3142–0.6732	0.0013	0.3785	0.1693–0.7472	0.0393*
	III–IV	331(214.4–391.1)						
Vascular	Present	472(370.8–481.4)	0.3713	0.2536–0.6834	0.0063	0.1843	0.1492–0.4951	0.0837
invasion	absent	325(209.8–390.0)						
Intratumor:	low/high^1^							
Foxp3^+^ Cells	Low	637(504.4–667.6)	0.3414	0.2511–0.4963	<0.0001	0.3351	0.2215–0.5457	0.0001*
	high	289(227.1–325.9)						
CD4^+^ T cells	Low	342(279.3–399.3)	0.9647	0.6735–1.384	0.8235	0.8336	0.6146–1.2483	0.7257
	high	531(398.4–563.5)						
CD8^+^ T cells	Low	176(127.5–225.8)	0.5643	0.3957–0.8046	0.0016	0.7582	0.5357–1.063	0.5369
	high	393(296.8–424.6)						

1 Two groups were divided by the median value. Excluded the special histologic type. (23 cases).

Abbreviations: HR, hazard ratio; 95% CI, 95% confidence interval; MST, median survival time.

*Significant.

## Discussion

TILs are a key component of the host immune response to cancer in the tumor microenvironment. Tregs potentially affect the TILs found in several types of human tumors, thus impairing cell-mediated immunity and promoting disease progression [32]–[34]. Increased percentages of CD4^+^CD25^+^ Tregs in PBMCs have been reported in patients with several types of human cancer [35], [36], including pancreatic cancer [30]. However, using FCM, we did not detect a significant difference in the relative abundance of CD4^+^CD25^+^Foxp3^+^ Tregs in the blood of PDA patients versus healthy controls. We speculate that this discrepancy in our results have been attributable to the definition of Treg cells. It has known that the activated T cells also express CD25, thus CD4 and CD25 double positive T cells probably do not represent the authentic Treg cells that should express the signature transcription factor Foxp3. Therefore, the CD4^+^CD25^+^Foxp3^+^ Treg frequency in this study should reflect the real Treg level in PDA patients, which is not significantly different from that in healthy control. These results also suggest the definition of Treg cell should be used with caution, which otherwise might cause the contradictory results as described above.

Though there is no apparent difference of Treg cells in PDA patients from that in controls, we found that the frequency of CD4^+^CD25^+^Foxp3^+^ Tregs was significantly higher in PDA tissues compared to the healthy pancreatic tissues. Moreover, using IHC, we also detected a high density of intratumoral FoxP3^+^ Tregs in PDAs, similar to results observed in other malignancies, including breast, ovarian, gastric and esophageal cancers [37]–[39]. This finding indicates that Tregs are significantly enriched in PDA tissues relative to the healthy pancreatic tissue, suggesting that Tregs are recruited to and remain in the PDA microenvironment to function. Due to the technical limitation, the IHC results could not reflect the real frequency of Treg cells in PDA tissues, which needs multiple color labeling technique. In this study, we firstly accurately defined the proportion of T cell subtypes including Treg cells in PDA tissue by FCM. The results further confirmed that the Treg frequency in the pancreatic tissues was much higher than that in healthy control.

We also found that the CD8^+^ T cell percentage was up-regulated in the PBMCs of PDA patients compared to PDA tissues and control PBMCs. Additionally, the relative intratumoral Treg and intratumoral CD8^+^ T cell abundances were negatively correlated in tumor tissue. These results are consistent with those found in other types of tumor tissues [40]. These findings indicate that a high proportion of intratumoral Tregs exists in PDA tissues, while CD8^+^ T cell recruitment is blocked. As such, by FCM, we evaluated correlations of the frequency of CD8^+^ T cells and CD4^+^CD25^+^Foxp3^+^ Tregs in PDA tissue with clinicopathologic characteristics. The findings suggest that the increased number of intratumoral Tregs was just only correlated with the tumor grade. However, by using IHC strategy, other researchers reported that the increased number of intratumoral Tregs were correlated with tumor status, pathologic metastasis status [27] and venous invasion [28]. These findings were consistent with our results that there were good correlation between tumor-infiltrating Foxp3^+^ cells and the clinicopathologic characteristics of 160 patients with PDA by using immunohistochemical staining (**Table S1**). To our knowledge, the activated CD4^+^T cells that transiently express Foxp3 may differentiate into memory T effector cells [41]. Therefore, using Foxp3 as the sole marker of Tregs may lead to discordant conclusions, and multistaining FCM assays might provide more accurate Treg frequency in tumor tissues than IHC-based methodology. Taken together, our observations of high proportions of CD4^+^CD25^+^FoxP3^+^ Tregs in tumors and CD8^+^ T cell recruitment blockage strongly support the possibility that these Tregs may inhibit local antitumor immunity at the tumor site. Tregs isolated from the tumor region can suppress autologous CD8^+^ T cell proliferation in vitro [42]. It is suggested that the local Treg abundance was achieved by suppressing the proliferation or migration of CD8^+^ T cells in PDA tissue. Results also suggested that, as the Treg cell population is the major suppressor in tumor immunity, targeting this sub-population rather than whole T cell population may represent a promising method for tumor immunotherapy.

A high intratumoral Treg density creates a generalized immunosuppressive microenvironment and contributes to tumor cell escape from immune surveillance [43]. The present study revealed high Foxp3^+^ cell numbers and a low-density CD8^+^ cells in the juxtatumoral stroma. However, the tumor stroma and normal pancreatic tissues did not show a high Foxp3^+^ cell prevalence. Our findings suggest that a higher number of tumor-infiltrating CD8^+^ cells were associated with a good survival but the PDA tumor microenvironment can recruit large numbers of functional Tregs, which can locally suppress the tumor-specific T-cell response. We conclude that the large Treg presence in the juxtatumoral stroma leads the tumor cells to evade the immune response, and the lack of low Treg numbers in the panstroma results in greater inflammation that promotes tumor invasion. Other studies have shown that Treg depletion results in enhanced antitumor responses and inhibits tumor growth [44], [45]. Furthermore, Treg-depleted CD4^+^ T cell transfer markedly augmented CD8^+^ T cell antitumor immune responses [11]. Cyclophosphamide administration, which preferentially removes CD4^+^CD25^+^ Tregs but not effector T cells, activated a latent pool of high-avidity tumor antigen-specific CD8^+^ T cells [46], [47]. These study results are consistent with our findings and are relevant for guiding immunotherapy targeting PDA.

In conclusion, our data demonstrate that the frequency of CD4^+^CD25^+^Foxp3^+^ Tregs is significantly increased in PDA tissue and that these cells inhibit tumor-associated antigen-specific CD8^+^ T cells. Furthermore, the increased tumor-infiltrating Treg abundance is positively correlated with PDA tumor cell differentiation and indicates a poor prognosis. The majority of Foxp3^+^ cells were found local to poorly differentiated PDA tumor cells. This observation is supported by our finding that the poorly differentiated PDA microenvironment recruits a high number of intratumoral Tregs, leading to a tumor cell immune evasion response and low survival rate among PDA patients. The panstroma exhibits a relatively low Treg abundance, which induces an inflammatory response in the stroma of the PDA microenvironment. Future studies should further explore the attenuation of Tregs around poorly differentiated PDA tumor cells; it is possible that the cytotoxic T cells and high density of Tregs observed in the juxtatumoral stroma that migrate to the panstroma can lead to diminished PDA tumor cells invasion.

## Supporting Information

Table S1 **Verification of the correlation between tumor-infiltrating Foxp3^+^ T cells and the clinicopathologic characteristics of 160 patients with PDA via immunohistochemical staining.**
(DOCX)

## References

[1] Raimondi S, Maisonneuve P, Lowenfels AB (2009) Epidemiology of pancreatic cancer: an overview. Nat Rev Gastroenterol Hepatol 6: 699–708. 19806144 10.1038/nrgastro.2009.177

[2] Kelsen DP, Portenoy R, Thaler H, Tao Y, Brennan M (1997) Pain as a predictor of outcome in patients with operable pancreatic carcinoma. Surgery 122: 53–59. 9225915 10.1016/s0039-6060(97)90264-6

[3] Ueno H, Schmitt N, Klechevsky E, Pedroza-Gonzalez A, Matsui T, et al (2010) Harnessing human dendritic cell subsets for medicine. Immunol Rev 234: 199–212. 20193020 10.1111/j.0105-2896.2009.00884.x PMC2847489

[4] Hiraoka N (2010) Tumor-infiltrating lymphocytes and hepatocellular carcinoma: molecular biology. Int J Clin Oncol 15: 544–551. 20924634 10.1007/s10147-010-0130-1

[5] de Vos van Steenwijk PJ, Ramwadhdoebe TH, Goedemans R, Doorduijn EM, van Ham JJ, et al (2013) Tumor-infiltrating CD14-positive myeloid cells and CD8-positive T-cells prolong survival in patients with cervical carcinoma. Int J Cancer 133: 2884–2894. 23740735 10.1002/ijc.28309

[6] Preston CC, Maurer MJ, Oberg AL, Visscher DW, Kalli KR, et al (2013) The Ratios of CD8(+) T Cells to CD4(+)CD25(+) FOXP3(+) and FOXP3(−) T Cells Correlate with Poor Clinical Outcome in Human Serous Ovarian Cancer. PLoS One 8: e80063. 24244610 10.1371/journal.pone.0080063 PMC3828213

[7] Ene-Obong A, Clear AJ, Watt J, Wang J, Fatah R, et al (2013) Activated pancreatic stellate cells sequester CD8+ T cells to reduce their infiltration of the juxtatumoral compartment of pancreatic ductal adenocarcinoma. Gastroenterology 145: 1121–1132. 23891972 10.1053/j.gastro.2013.07.025 PMC3896919

[8] Drake CG, Jaffee E, Pardoll DM (2006) Mechanisms of immune evasion by tumors. Adv Immunol 90: 51–81. 16730261 10.1016/S0065-2776(06)90002-9

[9] Campbell DJ, Koch MA (2011) Phenotypical and functional specialization of FOXP3+ regulatory T cells. Nat Rev Immunol 11: 119–130. 21267013 10.1038/nri2916 PMC3289970

[10] Kobayashi N, Hiraoka N, Yamagami W, Ojima H, Kanai Y, et al (2007) FOXP3+ regulatory T cells affect the development and progression of hepatocarcinogenesis. Clin Cancer Res 13: 902–911. 17289884 10.1158/1078-0432.CCR-06-2363

[11] Antony PA, Piccirillo CA, Akpinarli A, Finkelstein SE, Speiss PJ, et al (2005) CD8+ T cell immunity against a tumor/self-antigen is augmented by CD4+ T helper cells and hindered by naturally occurring T regulatory cells. J Immunol 174: 2591–2601. 15728465 10.4049/jimmunol.174.5.2591 PMC1403291

[12] Zou W (2006) Regulatory T cells, tumour immunity and immunotherapy. Nat Rev Immunol 6: 295–307. 16557261 10.1038/nri1806

[13] Curiel TJ (2007) Tregs and rethinking cancer immunotherapy. J Clin Invest 117: 1167–1174. 17476346 10.1172/JCI31202 PMC1857250

[14] Huang X, Wilber A, McIvor RS, Zhou X (2009) DNA transposons for modification of human primary T lymphocytes. Methods Mol Biol 506: 115–126. 19110623 10.1007/978-1-59745-409-4_9

[15] Bergmann C, Strauss L, Wang Y, Szczepanski MJ, Lang S, et al (2008) T regulatory type 1 cells in squamous cell carcinoma of the head and neck: mechanisms of suppression and expansion in advanced disease. Clin Cancer Res 14: 3706–3715. 18559587 10.1158/1078-0432.CCR-07-5126 PMC3708468

[16] Mougiakakos D, Choudhury A, Lladser A, Kiessling R, Johansson CC (2010) Regulatory T cells in cancer. Adv Cancer Res 107: 57–117. 20399961 10.1016/S0065-230X(10)07003-X

[17] Nishikawa H, Sakaguchi S (2010) Regulatory T cells in tumor immunity. Int J Cancer 127: 759–767. 20518016 10.1002/ijc.25429

[18] Cambien B, Karimdjee BF, Richard-Fiardo P, Bziouech H, Barthel R, et al (2009) Organ-specific inhibition of metastatic colon carcinoma by CXCR3 antagonism. Br J Cancer 100: 1755–1764. 19436305 10.1038/sj.bjc.6605078 PMC2695685

[19] Clark RA, Huang SJ, Murphy GF, Mollet IG, Hijnen D, et al (2008) Human squamous cell carcinomas evade the immune response by down-regulation of vascular E-selectin and recruitment of regulatory T cells. J Exp Med 205: 2221–2234. 18794336 10.1084/jem.20071190 PMC2556796

[20] Siddiqui SA, Frigola X, Bonne-Annee S, Mercader M, Kuntz SM, et al (2007) Tumor-infiltrating Foxp3-CD4+CD25+ T cells predict poor survival in renal cell carcinoma. Clin Cancer Res 13: 2075–2081. 17404089 10.1158/1078-0432.CCR-06-2139

[21] Clarke SL, Betts GJ, Plant A, Wright KL, El-Shanawany TM, et al (2006) CD4+CD25+FOXP3+ regulatory T cells suppress anti-tumor immune responses in patients with colorectal cancer. PLoS One 1: e129. 17205133 10.1371/journal.pone.0000129 PMC1762416

[22] Betts G, Jones E, Junaid S, El-Shanawany T, Scurr M, et al (2012) Suppression of tumour-specific CD4(+) T cells by regulatory T cells is associated with progression of human colorectal cancer. Gut 61: 1163–1171. 22207629 10.1136/gutjnl-2011-300970 PMC3388728

[23] Pang YL, Zhang HG, Peng JR, Pang XW, Yu S, et al (2009) The immunosuppressive tumor microenvironment in hepatocellular carcinoma. Cancer Immunol Immunother 58: 877–886. 18941744 10.1007/s00262-008-0603-5 PMC11030619

[24] Gobert M, Treilleux I, Bendriss-Vermare N, Bachelot T, Goddard-Leon S, et al (2009) Regulatory T cells recruited through CCL22/CCR4 are selectively activated in lymphoid infiltrates surrounding primary breast tumors and lead to an adverse clinical outcome. Cancer Res 69: 2000–2009. 19244125 10.1158/0008-5472.CAN-08-2360

[25] Pedroza-Gonzalez A, Xu K, Wu TC, Aspord C, Tindle S, et al (2011) Thymic stromal lymphopoietin fosters human breast tumor growth by promoting type 2 inflammation. J Exp Med 208: 479–490. 21339324 10.1084/jem.20102131 PMC3058586

[26] Labidi-Galy SI, Sisirak V, Meeus P, Gobert M, Treilleux I, et al (2011) Quantitative and functional alterations of plasmacytoid dendritic cells contribute to immune tolerance in ovarian cancer. Cancer Res 71: 5423–5434. 21697280 10.1158/0008-5472.CAN-11-0367

[27] Hiraoka N, Onozato K, Kosuge T, Hirohashi S (2006) Prevalence of FOXP3+ regulatory T cells increases during the progression of pancreatic ductal adenocarcinoma and its premalignant lesions. Clin Cancer Res 12: 5423–5434. 17000676 10.1158/1078-0432.CCR-06-0369

[28] Ino Y, Yamazaki-Itoh R, Shimada K, Iwasaki M, Kosuge T, et al (2013) Immune cell infiltration as an indicator of the immune microenvironment of pancreatic cancer. Br J Cancer 108: 914–923. 23385730 10.1038/bjc.2013.32 PMC3590668

[29] Vizio B, Novarino A, Giacobino A, Cristiano C, Prati A, et al (2012) Potential plasticity of T regulatory cells in pancreatic carcinoma in relation to disease progression and outcome. Exp Ther Med 4: 70–78. 23060925 10.3892/etm.2012.553 PMC3460315

[30] Yamamoto T, Yanagimoto H, Satoi S, Toyokawa H, Hirooka S, et al (2012) Circulating CD4+CD25+ regulatory T cells in patients with pancreatic cancer. Pancreas 41: 409–415. 22158072 10.1097/MPA.0b013e3182373a66

[31] Clark CE, Hingorani SR, Mick R, Combs C, Tuveson DA, et al (2007) Dynamics of the immune reaction to pancreatic cancer from inception to invasion. Cancer Res 67: 9518–9527. 17909062 10.1158/0008-5472.CAN-07-0175

[32] Lin YC, Mahalingam J, Chiang JM, Su PJ, Chu YY, et al (2013) Activated but not resting regulatory T cells accumulated in tumor microenvironment and correlated with tumor progression in patients with colorectal cancer. Int J Cancer 132: 1341–1350. 22907255 10.1002/ijc.27784

[33] Loddenkemper C, Hoffmann C, Stanke J, Nagorsen D, Baron U, et al (2009) Regulatory (FOXP3+) T cells as target for immune therapy of cervical intraepithelial neoplasia and cervical cancer. Cancer Sci 100: 1112–1117. 19514119 10.1111/j.1349-7006.2009.01153.x PMC11159425

[34] Pedroza-Gonzalez A, Verhoef C, Ijzermans JN, Peppelenbosch MP, Kwekkeboom J, et al (2013) Activated tumor-infiltrating CD4+ regulatory T cells restrain antitumor immunity in patients with primary or metastatic liver cancer. Hepatology 57: 183–194. 22911397 10.1002/hep.26013

[35] Ling KL, Pratap SE, Bates GJ, Singh B, Mortensen NJ, et al (2007) Increased frequency of regulatory T cells in peripheral blood and tumour infiltrating lymphocytes in colorectal cancer patients. Cancer Immun 7: 7. 17388261 PMC2935744

[36] Okita R, Saeki T, Takashima S, Yamaguchi Y, Toge T (2005) CD4+CD25+ regulatory T cells in the peripheral blood of patients with breast cancer and non-small cell lung cancer. Oncol Rep 14: 1269–1273. 16211295

[37] Curiel TJ, Coukos G, Zou L, Alvarez X, Cheng P, et al (2004) Specific recruitment of regulatory T cells in ovarian carcinoma fosters immune privilege and predicts reduced survival. Nat Med 10: 942–949. 15322536 10.1038/nm1093

[38] Ichihara F, Kono K, Takahashi A, Kawaida H, Sugai H, et al (2003) Increased populations of regulatory T cells in peripheral blood and tumor-infiltrating lymphocytes in patients with gastric and esophageal cancers. Clin Cancer Res 9: 4404–4408. 14555512

[39] Liyanage UK, Moore TT, Joo HG, Tanaka Y, Herrmann V, et al (2002) Prevalence of regulatory T cells is increased in peripheral blood and tumor microenvironment of patients with pancreas or breast adenocarcinoma. J Immunol 169: 2756–2761. 12193750 10.4049/jimmunol.169.5.2756

[40] Huang Y, Wang FM, Wang T, Wang YJ, Zhu ZY, et al (2012) Tumor-infiltrating FoxP3+ Tregs and CD8+ T cells affect the prognosis of hepatocellular carcinoma patients. Digestion 86: 329–337. 23207161 10.1159/000342801

[41] Zhou X, Bailey-Bucktrout SL, Jeker LT, Penaranda C, Martinez-Llordella M, et al (2009) Instability of the transcription factor Foxp3 leads to the generation of pathogenic memory T cells in vivo. Nat Immunol 10: 1000–1007. 19633673 10.1038/ni.1774 PMC2729804

[42] Thornton AM, Shevach EM (1998) CD4+CD25+ immunoregulatory T cells suppress polyclonal T cell activation in vitro by inhibiting interleukin 2 production. J Exp Med 188: 287–296. 9670041 10.1084/jem.188.2.287 PMC2212461

[43] de Rezende LC, Silva IV, Rangel LB, Guimaraes MC (2010) Regulatory T cell as a target for cancer therapy. Arch Immunol Ther Exp (Warsz) 58: 179–190. 20373146 10.1007/s00005-010-0075-0

[44] Dannull J, Su Z, Rizzieri D, Yang BK, Coleman D, et al (2005) Enhancement of vaccine-mediated antitumor immunity in cancer patients after depletion of regulatory T cells. J Clin Invest 115: 3623–3633. 16308572 10.1172/JCI25947 PMC1288834

[45] Litzinger MT, Fernando R, Curiel TJ, Grosenbach DW, Schlom J, et al (2007) IL-2 immunotoxin denileukin diftitox reduces regulatory T cells and enhances vaccine-mediated T-cell immunity. Blood 110: 3192–3201. 17616639 10.1182/blood-2007-06-094615 PMC2200901

[46] Ercolini AM, Ladle BH, Manning EA, Pfannenstiel LW, Armstrong TD, et al (2005) Recruitment of latent pools of high-avidity CD8(+) T cells to the antitumor immune response. J Exp Med 201: 1591–1602. 15883172 10.1084/jem.20042167 PMC2212915

[47] Lutsiak ME, Semnani RT, De Pascalis R, Kashmiri SV, Schlom J, et al (2005) Inhibition of CD4(+)25+ T regulatory cell function implicated in enhanced immune response by low-dose cyclophosphamide. Blood 105: 2862–2868. 15591121 10.1182/blood-2004-06-2410

