# Supplementary material for: An Increased Abundance of Tumor-Infiltrating Regulatory T Cells Is Correlated with the Progression and Prognosis of Pancreatic Ductal Adenocarcinoma
Source: PLoS One. 2014 Mar 17;9(3):e91551. doi: 10.1371/journal.pone.0091551 (PMC3956642; doi:10.1371/journal.pone.0091551)
Supplement: Table S1 — Verification of the correlation between tumor-infiltrating Foxp3+ T cells and the clinicopathologic characteristics of 160 patients with PDA via immunohistochemical staining. (DOCX) [file pone.0091551.s001.docx]

**Table S1. Verification of the correlation between tumor-infiltrating Foxp3^+^ T cells and the clinicopathologic characteristics of 160 patients with PDA via immunohistochemical staining**

| Characteristics | Intratumor Foxp3^+^ T cells | | |  | |
| --- | --- | --- | --- | --- | --- |
|  | Low(n=80) | High(n=80) | P |  | |
| Sex |  |  |  | |  |
| Male | 59（73.6） | 51（63.8） |  | |  |
| Female | 21（26.4） | 29（26.2） | 0.172 | |  |
| Mean age ± SD, years | 58.32±1.136 | 58.93±1.131 | --- | |  |
| Pathologic tumor status |  |  |  | |  |
| pT1 | 21(26.3) | 7(8.8) |  | |  |
| pT2 | 32(40.0) | 35(43.8) |  | |  |
| pT3 | 24(30.0) | 32(40.0) |  | |  |
| pT4 | 3(3.7) | 6(7.5) | 0.026 | |  |
| Pathologic metastasis status |  |  |  | |  |
| M0 | 76(95.0) | 73(91.3) |  | |  |
| M1 | 4(5.0) | 7(8.7) | 0.349 | |  |
| Pathologic node status |  |  |  | |  |
| N0 | 69(86.3) | 55(68.7) |  | |  |
| N1 | 11(13.7) | 25(21.3) | 0.008 | |  |
| Stage |  |  |  | |  |
| I+II | 72(90.0) | 52(65.0) |  | |  |
| III+IV | 8(10.0) | 18(25.0) | 0.011 | |  |
| Tumor Grade |  |  |  | |  |
| 1 | 28(35.0) | 8(10.0) |  | |  |
| 2 | 45(56.3) | 42(52.5) |  | |  |
| 3 | 7(8.7) | 30 (37.5) | 0.000 | |  |
| Vascular invasion |  |  |  | |  |
| Present | 10(12.5) | 25(31.2) |  | |  |
| Absent | 70(87.5) | 55(68.8) | 0.004 | |  |
| Lymphatic invasion |  |  |  | |  |
| Present | 7(8.8) | 12(15.0) |  | |  |
| Absent | 73(91.2) | 68 (75.0) | 0.222 | |  |
| ^1^χ^2^ test or Fisher’s exact test; ^2^Student’s t test. MV = median value. | | | | | |
| Classified according to International Union Against Cancer tumor-node-metastasis classification. | | | | | |
| Two groups were divided by the median value. Excluded the special histologic type. (23 cases) | | | | | |
